# Upregulation of Cysteine Protease Cathepsin X in the 6-Hydroxydopamine Model of Parkinson’s Disease

**DOI:** 10.3389/fnmol.2018.00412

**Published:** 2018-11-02

**Authors:** Anja Pišlar, Larisa Tratnjek, Gordana Glavan, Marko Živin, Janko Kos

**Affiliations:** ^1^Department of Pharmaceutical Biology, Faculty of Pharmacy, University of Ljubljana, Ljubljana, Slovenia; ^2^Institute of Pathophysiology, Medical Faculty, University of Ljubljana, Ljubljana, Slovenia; ^3^Department of Biology, Biotechnical Faculty, University of Ljubljana, Ljubljana, Slovenia; ^4^Department of Biotechnology, Jožef Stefan Institute, Ljubljana, Slovenia

**Keywords:** 6-hydroxydopamine, cathepsin X, dopaminergic neurons, glial cells, neurodegeneration, Parkinson’s disease

## Abstract

Parkinson’s disease (PD) is a neurodegenerative disorder characterized by loss of midbrain dopaminergic neurons in the substantia nigra pars compacta (SNc). *In vitro*, a contribution to neuroinflammation and neurotoxicity has been shown for the lysosomal protease cathepsin X; however, its expression and its role in PD remain unknown. Therefore, the current study was designed to address the regional, cellular, and subcellular localization and activity of cathepsin X in hemi-parkinsonian rats with 6-hydroxydopamine (6-OHDA)-induced excitotoxicity in the unilateral medial forebrain bundle (MFB) lesion. We report for the first time that cathepsin X expression and activity are rapidly increased in the ipsilateral SNc after injection of 6-OHDA into the MFB reaching a maximum after 12 h but seem to stay strongly upregulated after 4 weeks after injection. At early time points of 6-OHDA injection into the MFB, the increased cathepsin X is localized in the lysosomes in the neuronal, predominantly tyrosine hydroxylase-positive dopaminergic cells. After 12 h of 6-OHDA induced lesion, only a few activated microglial cells are positive for cathepsin X whereas, in 4 weeks post-lesion accompanied with complete loss of dopaminergic neurons, there is persistent cathepsin X upregulation restricted to activated glia cells. Taken together, our results demonstrate that cathepsin X upregulation in the lesioned dopaminergic system may play a role as a pathogenic factor in PD. Moreover, inhibition of cathepsin X expression or activity may be useful in protecting the nigrostriatal dopaminergic projection in the PD.

## Introduction

Parkinson’s disease is an age-related neurodegenerative disease with a primary pathology characterized by a progressive degeneration of the dopaminergic (DA) neurons of the substantia nigra pars compacta (SNc) (Qin et al., 2007). Although the precise etiology of chronic and progressive loss of DA neuron is still unclear, emerging evidence demonstrates that the inflammatory responses manifested by glia reactions, T cell proliferation, and increased expression of inflammatory cytokines, as well other toxic mediators derived from activated glial cells, are currently recognized as prominent features of Parkinson’s disease (PD) (Tufekci et al., 2012). Microglia – the resident immune cells of the central nervous system (CNS) – are involved in a plethora of neurodegenerative pathologies and, interestingly, show a higher density in the SNc and striatum than in other brain areas (Prinz and Priller, 2014; Haas et al., 2016). Microglial cells, also known as phagocytes of the brain, may be the main contributors to neuronal loss in PD, since they can produce large numbers of superoxide anions and other neurotoxins (McGeer and McGeer, 2008). In addition to inflammatory cytokines, activated microglia also secrete cathepsins, which may also promote neurodegeneration and contribute to neuronal loss in PD (Pislar and Kos, 2014).

Cysteine cathepsins, lysosomal peptidases, constitute the largest cathepsin family, comprising 11 members (cathepsins B, C, F, H, K, L, O, S, W, V, and X), and possess a conserved active site involving cysteine, histidine, and asparagine residues in a catalytic triad (Rawlings et al., 2012). They are primarily responsible for terminal protein degradation in lysosomes; however, their role in regulating a number of other important physiological and pathological processes has been demonstrated (Obermajer et al., 2006; Kos et al., 2009; Pislar and Kos, 2014). Recent studies have suggested that cathepsin X is an important player in degenerative processes during normal aging and in neurodegenerative diseases. It’s expression and proteolytic activity are strongly upregulated in mouse brain, with a preference for glial cells and aged neurons, particularly in a transgenic mouse model of Alzheimer’s disease (AD) in which cathepsin X upregulation in microglial cells surrounding amyloid plaques has been observed (Wendt et al., 2007; Hafner et al., 2013). The contribution of cathepsin X to amyloid-β-related neurodegeneration also is exerted through proteolytic cleavage of the C-terminal dipeptide of γ-enolase, abolishing its neurotrophic and neuroprotective activity (Hafner et al., 2013; Pislar and Kos, 2013). Besides carboxypeptidase activity, cathepsin X activity in its pro-form has also been demonstrated (Nagler and Menard, 1998; Santamaria et al., 1998). In addition to a role in neurodegeneration, the involvement of cathepsin X in inflammation-induced neurodegeneration has been demonstrated (Stichel and Luebbert, 2007; Wendt et al., 2009; Pišlar et al., 2017). Moreover, we earlier provided evidence for the participation of cathepsin X in the apoptosis of neuronal cells induced by 6-hydroxydopamine (6-OHDA), using a cell model that mimics several pathological features of PD. The involvement of cathepsin X in neurotoxin-induced cell death was further confirmed with a specific inhibitor of cathepsin X that protects neuronal cells against 6-OHDA toxicity, indicating that cathepsin X may promote the pathogenic cascade event in PD (Pislar et al., 2014). However, the expression of cathepsin X in the brain of PD patients or in animal models of PD has not been studied.

In the present study, we have investigated the expression pattern and cellular localization of lysosomal cysteine cathepsin X in the rat brain following the unilateral, 6-OHDA–induced lesion in the MFB. Cathepsin X was found to be markedly upregulated in the ipsilateral SNc with a concomitant reduction of dopaminergic neurons. Increased expression of cathepsin X in the ipsilateral SNc was predominantly localized to lysosomes in dopaminergic neurons. However, after an extended 6-OHDA-induced lesion, upregulated cathepsin X was localized only in activated microglia cells. Our results suggest that elevated cathepsin X expression and its activity is implicated in neurodegeneration in the nigrostriatal DA system involved in PD.

## Materials and Methods

### Animals

Male Wistar rats, from the Medical Experimental Center, Medical Faculty, University of Ljubljana, Slovenia, weighing 280–350 g (7–9 weeks old) at the beginning of the experiments were housed at 22 ± 2°C and 60 ± 10% relative humidity under a 12 h light/dark cycle, with free access to food and water. All animal-related procedures were conducted in accordance with the European Communities Council Directive of 2010 (2010/63/UE) for animal experiments and the National Veterinary Institute Guide for the Care and Use of Laboratory Animals. Care was taken to minimize the number of experimental animals and their suffering. The experiments were approved by the Administration of the Republic of Slovenia for Food Safety, Veterinary Sector, and Plant Protection.

### Unilateral 6-OHDA Lesions of the Nigrostriatal Pathway

Sixteen animals were anesthetized with an intraperitoneal injection of 2% xylazine hydrochloride (8 mg/kg; Rompun^®^; Bayer, Leverkusen, Germany) and 10% ketamine (28 mg/kg; Bioketan, Vetoquinol; Biowert, Gorzow, Poland) and were positioned in a stereotaxic frame (TrentWells, South Gate, CA, United States) with the incisor bar at the level of the ear. 6-OHDA hydrobromide (8 μg of free base dissolved in 0.9% saline containing 0.02% ascorbic acid; Sigma-Aldrich, MO, United States) was infused at a rate of 1 μl/min over 4 min into the right MFB at the following coordinates: anterior 4.4 mm from lambda, lateral 1.0 mm from the midline and ventral 7.8 mm from the surface of dura [stereotaxic coordinates (Torres and Dunnett, 2011)]. The cannula was left at the injection site for an additional 3 min post-injection before being slowly retracted.

### Brain Tissue Preparation

The rats were divided into four groups, sacrificed at different times, i.e., 12 h (*n* = 4), 24 h (*n* = 4), 48 h (*n* = 4), or 4 weeks (*n* = 4) following the injections of 6-OHDA. The brains were rapidly removed and quickly frozen on dry ice and stored at -80°C in a freezer until cryostat sections could be cut. Coronal sections (10–20 μm), identified using a rat brain atlas (Paxinos and Watson, 2007), were cut at four anterior-posterior levels through the striatum (in mm from bregma); (1) between 1.92 and 1.20, (2) between 1.08 and 0.24, (3) between 0.12 and -0.48, and (4) between -0.60 and -1.44. Sections were mounted on numbered serial glass slides in such a way that each glass slide contained one brain slice from the specific striatal subregion. Similarly, SNc was cut at three anterior–posterior levels (in mm from bregma); (1) between 4.68 and -5.04, (2) between -5.20 and -5.52, and (3) between -5.64 and -6.00 and mounted on glass slides (three slices of three SNc subregions). The sections were mounted onto microscope glass slides coated with a 0.01% solution of (poly)L-lysine (Sigma-Aldrich, St. Louis, MO, United States). The slides were then vacuum-packed and stored in a freezer at –20°C until being further processed. For the *in situ* hybridization histochemistry, immunohistochemistry, and double immunofluorescence staining, four 10 μm sections of the striatum and four 10 μm sections of the SNc from each animal were analyzed. For the protein expression and activity analysis, eight 20 μm sections of the SNc were used.

### Assessment of 6-OHDA-Induced Neuronal Loss

The extent of the nigrostriatal dopaminergic cell loss after 6-OHDA lesion was assessed by the tyrosine-hydroxylase (TH) mRNA *in situ* hybridization histochemistry and TH immunohistochemistry (see the protocols below). The coronal brain slices at the level of SNc were analyzed. Only animals with evident downregulation of TH mRNA and TH protein level in the ipsilateral SNc were used in subsequent analyses. Animals sacrificed 4 weeks after 6-OHDA injection were additionally tested for the development of nigrostriatal degeneration by apomorphine test. The 6-OHDA-lesioned animals were treated with directly acting mixed agonist of DA receptors apomorphine hydrochloride (0.05 mg/kg, s.c.; Sigma-Aldrich) in the fourth post operative week. The rats were placed in plastic cylindrical chambers (40 cm diameter), and the number of contralateral turning was counted manually. The animals showing >200 stereotyped contralateral turns per hour after a single dose of apomorphine were used in the subsequent experiments.

### *In situ* Hybridization Histochemistry

The standard procedure described by Zivin et al. (1996) was used for *in situ* hybridization histochemistry. The 10 μm sections were incubated with ^35^S-labeled oligodeoxyribonucleotide “antisense” probes (45 bases long) complementary to the rat TH mRNA (sequence 5^′^-AAC CAA ACC AGG GCA CAC AGG GAG AAC CAT GCT CTT AAG-3^′^) and rat cathepsin X mRNA (sequence 5^′^-AGG TCT CAT CGG GGA TGC CAT GCT TGT GGG CAT ACT CCC ACA CCG-3^′^). The GenBank accession numbers used to design the probes were TH M23598 and CTSX NM183330. Hybridized sections were exposed to X-ray film (Scientific Imaging Film X-OmatTM AR, Kodak, Rochester, NY, United States) for 2–3 weeks and developed using standard darkroom techniques.

### Emulsion Autoradiography

The slides from *in situ* hybridization were immersed in Ilford K2 emulsion in gel form (HARMAN technology Limited, Cheshire, England) and exposed for 9 weeks at 4°C in the dark before being developed with Kodak GBX developer (Sigma-Aldrich) (1:5, vol/vol) for 5 min, then fixed in Kodak GBX fixer (1:15, vol/vol, Sigma-Aldrich) for 10 min. After washing with water, the sections were counterstained with 0.2% methylene blue, dehydrated, coverslipped with DPX (Sigma-Aldrich), and photographed under a light microscope (Olympus IX81, Olympus Optical, Tokyo, Japan) with a digital camera (Olympus DP71).

### Semi-Quantification of *in situ* Hybridization Histochemistry

The *in situ* hybridization signal was semi-quantified using an image analyser software (ImageJ, National Institutes of Health, Bethesda, MD, United States). An intensity threshold was set based on the mean gray values measured within the cerebral peduncle. Mean gray value of the area covered by the above-threshold pixels in the manually outlined region was determined separately for the contralateral and ipsilateral side. In the graph, data are presented as means ± standard deviation (SD) determined from four animals. Differences between ipsilateral and contralateral sides within each experimental group were analyzed using the two-tailed paired Student’s *t*-test.

### Immunohistochemistry

Coronal brain sections (10 μm) were fixed in cold 100% methanol for 5 min followed by 15 min in cold methanol with 1% H_2_O_2_. Brain slices were incubated in blocking buffer containing 4% normal serum, 1% BSA, and 0.1% Triton X-100 in potassium phosphate buffer (KPBS) for 1 h at room temperature. They were then incubated overnight at 4°C with mouse monoclonal antibody against TH (1:750, Abcam, Cambridge, United Kingdom), goat polyclonal primary antibody against cathepsin X (1:200, AF934, R&D Systems, MN, United States) or mouse monoclonal antibody against OX-6 (1:300, Abcam, Cambridge, United Kingdom), diluted in blocking solution and then with biotinylated anti-mouse or anti-goat secondary antibodies (1:750, Vector Laboratories, Burlingame, CA, United States) diluted in KPBS containing 1% BSA and 0.02% Triton X-100 for 1.5 h at room temperature. Avidin–biotin–peroxidase complex (ABC elite standard kit, Vector Laboratories, Burlingame, CA, United States) was added for 30 min. Staining was visualized with 3,3′-diamino-benzidine (DAB, Sigma-Aldrich). All sections were immunolabeled simultaneously to ensure the same conditions, such as using identical DAB staining incubation times. Sections were then mounted, dehydrated, and coverslipped with DPX mounting medium (BDH Laboratory Supplies, Poole, United Kingdom). Brain sections were examined and imaged with an Olympus microscope (Olympus IX81) with an attached digital camera (Olympus DP71) using the same system settings for all samples.

### Protein Extraction

For analysis of the protein level of cathepsin X and its activity, the SNc was dissected out from eight 20 μm frozen brain slices of 6-OHDA rat brains, separately from the contralateral (Control) and ipsilateral side (Lesion), using a cryostat at -20°C. Tissue was homogenized in ice-cold lysis buffer (0.05 M sodium acetate, pH 5.5, 1 mM EDTA, 0.1 M NaCl, 0.25% Triton X-100) supplemented with a cocktail of phosphatase inhibitors (Thermo Fisher Scientific, Waltham, MA, United States), then sonicated and centrifuged at 15.000 *g* at 4°C for 15 min to collect the supernatant. Total protein concentration was determined by DC^TM^ Protein Assay (Bio-Rad, Hercules, CA, United States). All the samples were kept at -70°C until used for analysis.

### ELISA

The protein level of cathepsin X was determined by ELISA, as reported (Kos et al., 2005). Briefly, microtiter plates were coated with equal aliquots of goat anti-cathepsin X AF934 antibody (RD Systems) in 0.01 M carbonate/bicarbonate buffer, pH 9.6, at 4°C. After blocking with 2% BSA in PBS, pH 7.4, for 1 h at room temperature, the samples of equal protein amount (50 μg) or cathepsin X standards were added. Following 2 h incubation at 37°C, the wells were washed and filled with mouse anti-cathepsin X 3B10 monoclonal antibody conjugated with horseradish peroxidase in blocking buffer. After a further 2 h incubation at 37°C, 200 μl/well of 3,3′,5,5′-tetramethylbenzidine substrate (Sigma-Aldrich) in 0.012% H_2_O_2_ was added. After 15 min, the reaction was stopped by adding 50 μl of 2 μM H_2_SO_4_. The amount of protein was determined by measuring the absorbance at 450 nm using a microplate reader (Tecan Safire^2^, Tecan, Switzerland), and the concentration of cathepsin X was calculated from the calibration curve.

### Cathepsin X Activity

Cathepsin X activity was measured in tissue lysates with the cathepsin X-specific, intramolecularly quenched fluorogenic substrate Abz-Phe-Glu-Lys(Dnp)-OH synthesized by Jiangsu Vcare Pharmatech Co. (China). An aliquot of 50 μg of the lysate proteins was incubated at 37°C, followed by measurement of cathepsin X activity using 10 μM Abz-Phe-Glu-Lys(Dnp)-OH. The fluorometric reaction was quantified at 37°C at an excitation wavelength of 320 nm and emission wavelength of 420 nm on a microplate reader (Tecan Safire^2^). Results are presented as a change in fluorescence as a function of time (ΔF/Δt), and cathepsin X activity was expressed relative to Control.

### Double Immunofluorescence Labeling

The striatal and nigral brain sections (10 μm) of 6-OHDA treated rats were double-immunostained for cathepsin X localization analysis using the following primary antibodies: goat anti-cathepsin X (1:75, R&D System), the lysosomal marker, rabbit anti-LAMP1 (1:75, Sigma-Aldrich), the neuronal marker, mouse anti-NeuN (1:300, EMD Millipore, Billerica, MA, United States), the microglia marker, mouse anti-OX-6 (1: 300, Abcam), the astrocyte marker, mouse anti-GFAP (1:1000, Abcam), and the marker for dopaminergic neurons, mouse anti-TH (1:500, Abcam). The immunofluorescence procedure was performed as reported (Tratnjek et al., 2013). Briefly, brain slices were fixed in cold methanol for 20 min and then incubated in blocking solution containing 4% donkey serum (EMD Millipore) and 0.4% Triton X-100 in KPBS for 1 h at room temperature. Brain sections were then incubated with the primary antibodies overnight at 4°C diluted in KPBS containing 1% donkey serum and 0.4% Triton X-100 and followed by 1.5 h incubation at room temperature. Sections were then incubated with the Alexa-fluorophore-conjugated secondary antibodies (1:300, Invitrogen, Molecular Probes, OR, United States) diluted in KPBS containing 0.02% Triton X-100 for 1.5 h at room temperature. After incubation, sections were immersed in 0.1% Sudan Black B (Sigma-Aldrich) in 70% ethanol (vol/vol) for 5 min to suppress the lipofuscein autofluorescence background. Sections were then rinsed with KPBS, mounted on glass slides using the ProLong Gold Antifade Mountant with DAPI (Thermo Fisher Scientific). To confirm staining specificity, primary antibodies were omitted. Negative controls always yielded negative results (*data not shown*). Fluorescence microscopy was performed using a Carl Zeiss LSM 710 confocal microscope (Carl Zeiss, Oberkochen, Germany). Images were analyzed using Carl Zeiss ZEN 2011 image software.

### Statistical Analysis

Results of cathepsin X protein level and activity are representative of two independent experiments, each performed in duplicate, and are presented as means ± SD. ANOVA test and *post hoc* analysis for multiple comparisons using Dunnett’s multiple comparisons *t*-test using GraphPad Prism, version 6 was used for statistical evaluation when two sets of values were compared; *P* < 0.05 was considered to be statistically significant.

## Results

### Loss of TH-Positive Dopaminergic Midbrain Neuronal Nuclei After 6-OHDA Injection

**FIGURE 1:**
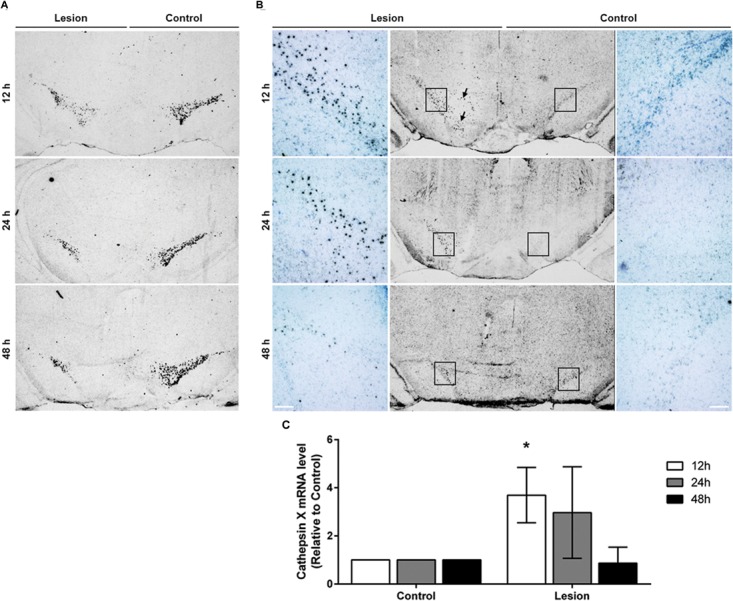
The expression pattern of TH and cathepsin X mRNA in the SNc after 6-OHDA injection. Representative *in situ* hybridization histochemistry images of coronal midbrain sections of the SNc from the 12, 24, and 48 h time points after 6-OHDA-induced lesion. **(A)** TH mRNA seen as clusters of silver grains, shows reduced TH mRNA expression in the ipsilateral SNc (Lesion) at all time points indicated, in comparison to the contralateral side (Control). **(B)** 6-OHDA-induced lesions at 12 and 24 h increased cathepsin X mRNA expression in the ipsilateral SNc (Lesion) compared to the contralateral side (Control). At 12 h, on the ipsilateral side, cathepsin X mRNA-positive signals were also observed in the VTA region (black arrow). At 48 h after 6-OHDA injection, the intensity and density of increased cathepsin X mRNA in the ipsilateral SNc were lower than those for cathepsin X mRNA signals at 12 and 24 h. For each condition, a group of four animals (*n* = 4) was analyzed and four sections of the SNc region of each animal were analyzed. *Scale bar*s = 200 μm. **(C)** Semi-quantification of cathepsin X mRNA expression. The graph represents the average of the *in situ* hybridization signal for cathepsin X of the ipsilateral SNc (Lesion) expressed relative to contralateral SNc (Control) from the 12, 24, and 48 h time-points after 6-OHDA-induced lesion. Values are means ± SD of group of four animals. ^∗^*P* < 0.05.

**FIGURE 2:**
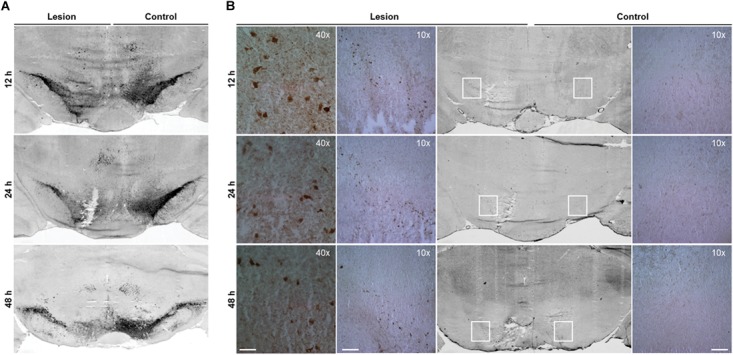
Increased cathepsin X immunoreactivity in the SNc after progressive degeneration of the nigral dopaminergic neurons. Representative immunohistochemical images of coronal midbrain sections of the SNc from the 12, 24, and 48 h time points after 6-OHDA-induced lesion. **(A)** TH staining demonstrates that the number of TH-positive neurons in the ipsilateral SNc is reduced by 6-OHDA injection (Lesion) at all time points indicated, in comparison to that on the contralateral side (Control). **(B)** Cathepsin X immunoreactivity in the ipsilateral SNc is increased markedly at 12 and 24 h after 6-OHDA injection (Lesion) compared to the contralateral side (Control). The immunoreactivity of cathepsin X gradually decreases over time; however, the increased expression of cathepsin X is still present 48 h after 6-OHDA injection. For each condition, a group of four animals (*n* = 4) was analyzed and four sections of the SNc region of each animal were analyzed. *Scale bars* = 200 μm (10×), 50 μm (40×).

*In vitro* study revealed that 6-OHDA induces the protein level and activity of cathepsin X in dopaminergic-like cells (Pislar et al., 2014). In order to elucidate the expression of cathepsin X during the 6-OHDA-induced lesion *in vivo*, male rats were unilaterally lesioned by an injection of 6-OHDA into the right MFB. The loss of dopaminergic neurons was evaluated in the SNc on coronal sections obtained at 12, 24, and 48 h after 6-OHDA injection by assessing the expression of TH mRNA, a dopaminergic neuronal marker. As shown in Figure 1A, there was a rapid loss of autoradiographic TH mRNA signals in the SNc on the 6-OHDA-induced ipsilateral side at 12 h. The loss of signals of TH mRNA continued up to 48 h after the lesion, as compared with those of the intact contralateral sides. Likewise, immunohistochemical analysis of the ipsilateral SNc showed gradually decreasing TH-immunoreactivity from 12 to 48 h after injection of 6-OHDA (Figure 2A). Further, the number of TH-positive neurons in the ventral tegmental area (VTA) in the ipsilateral SNc started to decrease; 48 h after injection less than half of the TH-positive VTA neurons were detectable compared to contralateral SNc. However, the staining intensities of TH-positive fibers in the striatum were similar in contralateral and ipsilateral sides, even at 48 h 6-OHDA injection (Supplementary Figure S1A). Together, these results indicate that the early time points of injection of 6-OHDA-induced neurodegeneration leads to rapid loss of TH-positive neurons in SNc; however, the intensity of the TH immunosignal in the striatum was not changed from 12 to 48 h after 6-OHDA injection.

### Increased Cathepsin X Expression in the 6-OHDA-Lesioned SNc

After the pattern of midbrain TH-positive neuron loss in rat induced by 6-OHDA was established, the time course of cathepsin X expression in the SNc was addressed. First, cathepsin X expression at the transcriptional level in the SNc, at 12, 24, and 48 h after 6-OHDA-induced lesion, was observed by *in situ* hybridization histochemistry and emulsion autoradiography. The results show a marked increase of signals for cathepsin X mRNA in the ipsilateral SNc at 12 h after the 6-OHDA injection relative to those in the contralateral SNc (Figure 1B, *upper panel*), and the upregulation of cathepsin X mRNA was observed also at the 24 h time point after the injection (Figure 1B, *middle panel*), whereas at 24 h the increase was not significant as was at 12 h after 6-OHDA-induced lesion (Figure 1C). However, the density of cathepsin X mRNA positive signals in the ipsilateral SNc was similar to the contralateral side (Figure 1B, lower panel). Additionally, 12 h after the 6-OHDA-induced lesion, the few cathepsin X mRNA positive signals observed on the ipsilateral side were scattered in the VTA region (Figure 1B). Further, we confirmed the upregulation of cathepsin X at the translation level by using immunohistochemical staining. These results showed that the expression pattern of cathepsin X protein was consistent with that of cathepsin X mRNA. Thus, as depicted in Figure 2B, there was a decreasing trend of cathepsin X upregulation in the ipsilateral SNc, compared to the appropriate contralateral SNc, from 12 to 48 h 6-OHDA injection. However, even at 48 h, cathepsin X immunoreactivity on the ipsilateral side differed substantially from that on the contralateral side, suggesting that cathepsin X expression in the SNc is rapidly upregulated upon 6-OHDA lesion, but appears to stay overexpressed at all time points analyzed. On the contrary, no obvious change in cathepsin X immunoreactivity was observed in the striatum at 12, 24, or 48 h after the 6-OHDA-induced lesion (Supplementary Figure S1B), and the similar expression intensity of cathepsin X on the contralateral and ipsilateral sides was consistent with that of TH (Supplementary Figure S1A).

**FIGURE 3:**
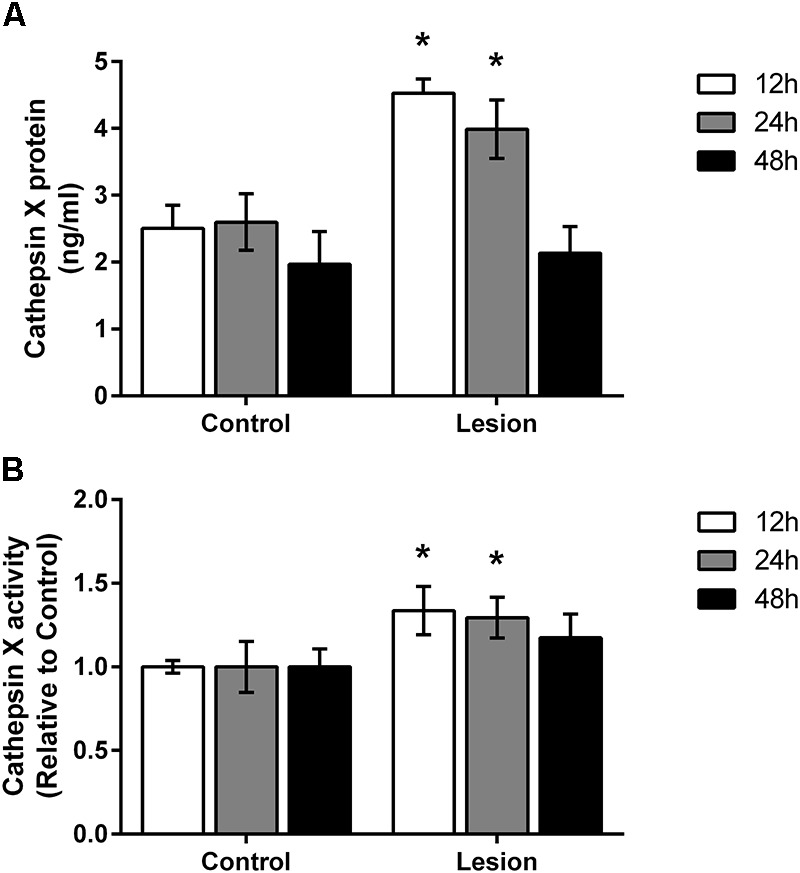
Time-dependent increase of cathepsin X protein level and activity in the SNc following 6-OHDA injection. **(A)** ELISA analysis of the cathepsin X protein level in the SNc dissected from the coronal midbrain sections from the 12, 24, and 48 h time-points after 6-OHDA-induced lesion. Cathepsin X protein levels in the ipsilateral SNc (Lesion) were significantly increased at 12 and 24 h after 6-OHDA injection compared to the contralateral side (Control), whereas no changes in protein expression were detected after 48 h injection. **(B)** Enzyme activity analysis shows an increase in cathepsin X activity in the ipsilateral SNc (Lesion) at 12 and 24 h after 6-OHDA-induced lesion, whereas after 48 h the increase in cathepsin X activity in the ipsilateral side was not significant compared to the contralateral side (Control). Values are means ± SD of group of four animals, each analyzed in duplicate.^∗^*P* < 0.05.

Based on the immunohistochemical observations of cathepsin X upregulation after 6-OHDA injections, proteins were isolated from the dissected contralateral and ipsilateral SNc regions of coronal sections in order to confirm changes in cathepsin X protein level and in its activity, using quantitative methods such as ELISA and enzymatic activity. As shown in Figure 3A, cathepsin X was significantly upregulated in the ipsilateral SNc compared to the contralateral side at 12 and 24 h time points of injection. Although a slight increase in cathepsin X level was also detectable in the ipsilateral SNc at 48 h compared with the level in the contralateral SNc, this increase did not reach statistical significance. Furthermore, analysis of protein activity showed that the 6-OHDA-induced lesion also increased cathepsin X activity in the ipsilateral SNc relative to contralateral at all time points analyzed. However, statistical analysis revealed significant differences in cathepsin X activity only at 12 and 24 h time points (Figure 3B), and the increase in cathepsin X activity was noticeable to a much smaller extent as an increase in protein level, indicating the presence of an increased proform of cathepsin X rather than the mature form. Nevertheless, analysis of enzyme activity in the striatum showed no differences in cathepsin X activity between contralateral and ipsilateral side after 6-OHDA injection (data not shown), which coincides with the results obtained by immunohistochemical staining. Collectively, these findings indicate that cathepsin X expression, and activity are increased in the SNc under short-term neurodegenerative conditions.

### 6-OHDA-Induced Upregulation of Cathepsin X in Dopaminergic Neurons

Intrigued by the fact that cathepsin X is a lysosomal peptidase, we first studied the vesicular localization of upregulated cathepsin X in the SNc after 6-OHDA injection. Double immunofluorescence staining with a lysosomal marker LAMP1 was performed. Results show the prominent co-localization of cathepsin X and LAMP1 formatted in vesicular structures in cells in the ipsilateral SNc after 6-OHDA injection at time points determined (Supplementary Figure S2).

**FIGURE 4:**
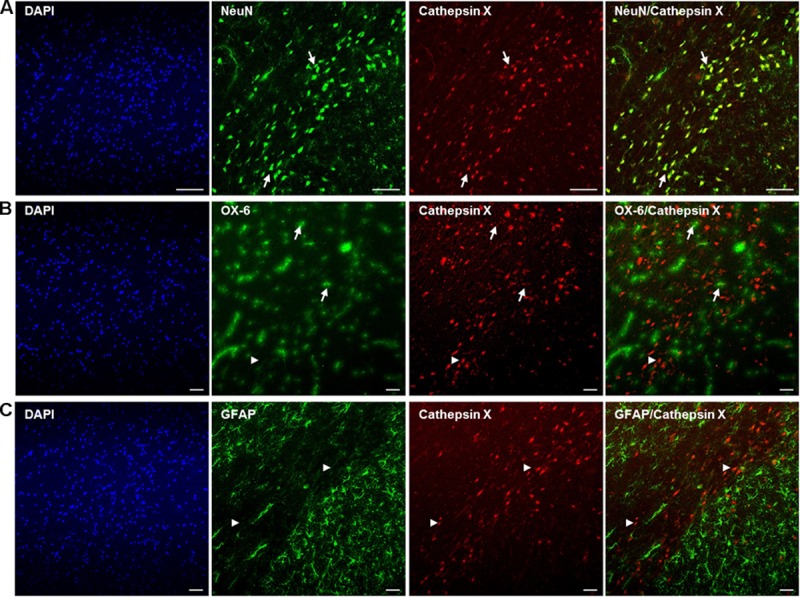
Phenotype of increased cathepsin X-immunopositive cells in the lesioned SNc after 6-OHDA injection. Representative images of double immunofluorescence staining of cathepsin X (red fluorescence) and cell-type markers (green fluorescence) for neurons (NeuN, **A**), microglial cells (OX-6, **B**) and astrocytes (GFAP, **C**) in the ipsilateral SNc at 12 h after the injection of 6-OHDA. Nuclei were counterstained with DAPI (blue fluorescence). Expression of cathepsin X in the lesioned SNc is predominantly restricted to NeuN-positive neuronal cells [**(A)** arrows]. A single cathepsin X-positive microglial cell in the SNc was notable (**B**, arrows), while most microglial cells were negative for cathepsin X (arrowheads). No astrocytes were positive for cathepsin X (**C**, arrowheads). Groups of four animals (*n* = 4) were analyzed and four sections of the SNc region of each animal were analyzed. *Scale bar* = 100 μm.

After detection of increased cathepsin X vesicular expression in the ipsilateral SNc, induced by 6-OHDA lesion, brain slices were double-immunofluorescently stained for cathepsin X and markers for neurons (NeuN), microglial cells (OX-6) and astrocytes (GFAP), to elucidate the cell type expressing increased cathepsin X in the degenerated SNc at the 12 h-induced lesion. The majority of cathepsin X-positive cells in the ipsilateral SNc expressed the neuronal marker NeuN (Figure 4A), while the microglial cells found in SNc were mostly negative for cathepsin X expression (Figure 4B). Reactive astrocytes surrounding SNc also did not display any cathepsin X immunoreactivity (Figure 4C).

**FIGURE 5:**
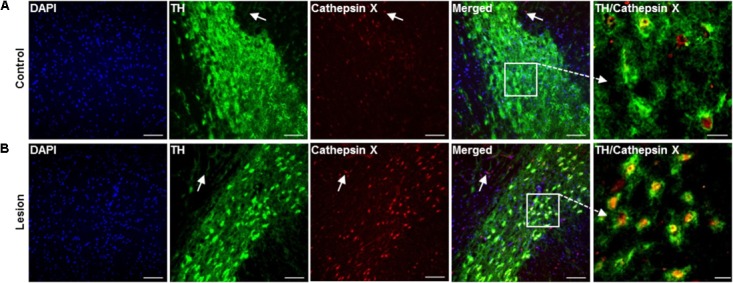
Increased expression of cathepsin X in TH-positive neurons in the SNc after 6-OHDA injection. Representative images of double immunofluorescence staining of TH (green fluorescence) and cathepsin X (red fluorescence) in the SNc at 12 h after the injection of 6-OHDA. Nuclei were counterstained with DAPI (blue fluorescence). In the contralateral SNc (Control), single TH-positive neuronal cells were cathepsin X-positive **(A)**, whereas cathepsin X was upregulated in the majority of TH-positive cells in the ipsilateral SNc (Lesion) **(B)**. Some surrounding TH-negative cells in contralateral and ipsilateral SNc were also cathepsin X-positive, as indicated by white arrows. Groups of four animals (*n* = 4) were analyzed and four sections of the SNc region of each animal were analyzed. *Scale bar* = 100 μm.

To confirm the upregulation of cathepsin X in dopaminergic neurons in the SNc at the short term-induced lesion, we performed additional double immunostaining with the dopaminergic neuronal marker TH, using brain slices at 12 h of 6-OHDA-induced lesion. As shown in Figure 5A, only a few TH-positive cells expressed cathepsin X in the contralateral SNc. However, the majority of cathepsin X-positive cells were expressing TH, and the number of TH-positive cells with increased cathepsin X expression was strongly elevated in the ipsilateral SNc (Figure 5B). Interestingly, in the vicinity of dopaminergic neuronal cells, cathepsin X expression in TH-negative cells was also observed, presumably due to its expression in other cells, such as microglial cells.

**FIGURE 6:**
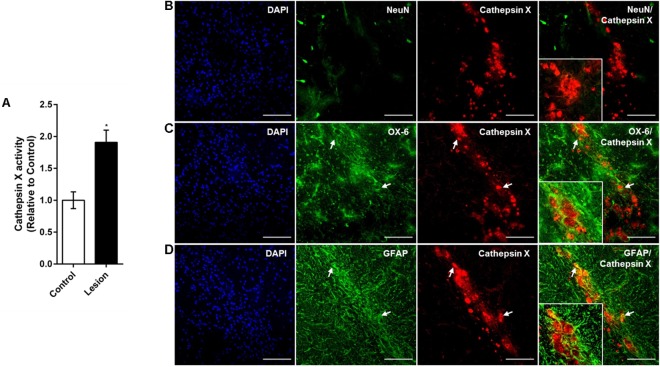
Phenotype of increased cathepsin X-immunopositive cells in the ipsilateral SNc after long-term 6-OHDA injection. **(A)** Enzymatic activity analysis of cathepsin X in the SNc, dissected from the coronal sections from the 4 weeks’ time-point after 6-OHDA-induced lesion. Significantly increased cathepsin X activity in the ipsilateral SNc (Lesion) was observed, compared to that in the contralateral side (Control). Values are means ± SD of four experiments. ^∗^*P* < 0.05 **(B–D)** Representative images of double immunofluorescence staining of cathepsin X (red fluorescence) and cell-type markers (green fluorescence) for of neurons (NeuN, **B**), microglial cells (OX-6, **C**) or astrocytes (GFAP, **D**) in the ipsilateral SNc at 4 weeks after the 6-OHDA injection. Nuclei were counterstained with DAPI (blue fluorescence). Neuronal cells surrounding the site of injection were negative for cathepsin X expression. Increased cathepsin X expression in ipsilateral SNc was predominantly restricted to microglial cells (**C**, white arrows) and some astrocytes (**D**, white arrows). Groups of four animals (*n* = 4) were analyzed and four sections of the SNc region of each animal were analyzed. *Scale bars* = 100 μm.

Notably, some single, cathepsin X-positive microglial cells were observed in the ipsilateral SNc at 12 h after the 6-OHDA-induced lesion (Figure 4B). The number of microglial cells expressing cathepsin X was higher at 48 h (Supplementary Figure S3), which correlates well with sustained microglia reactivity after a longer period of time of 6-OHDA injection. Moreover, 4 weeks after 6-OHDA lesion, cathepsin X level was still significantly increased, as indicated by increased cathepsin X activity in the ipsilateral SNc compared to the contralateral side (Figure 6A). Immunofluorescence staining also revealed strong upregulation of cathepsin X in the ipsilateral SNc; however, no NeuN-positive cells were observed at the lesioned SNc probably due to the complete loss of dopaminergic cells after 4 weeks of 6-OHDA injection (Figure 6B). Remarkably, upregulated cathepsin X was restricted to glia cells, where cathepsin X-positive microglia cells (Figure 6C) and cathepsin X-positive astrocytes (Figure 6D) were found, indicating an important role for cathepsin X in progressive inflammation related to chronic neurodegeneration.

## Discussion

The contribution of cathepsin X to neuroinflammation and neurotoxicity in *in vitro* models mimicking neurodegenerative processes has been demonstrated; however, its expression pattern and role in the brain with PD remained unknown. Therefore, in the present study, we characterized the regional and cellular expression, as well as the activity of cathepsin X in striatum and SNc of rat 6-OHDA model of PD, in a time course manner. Our results show for the first time that 6-OHDA, injected into the MFB, leads to an increased level of cathepsin X mRNA, as well as protein expression and its activity in the ipsilateral SNc. Upregulated cathepsin X has been at early time points localized in the lysosomes in neuronal cells, with preferential localization in dopaminergic neurons. Nevertheless, on the short-term, specifically at 12 h after 6-OHDA injection, the association of single-microglial cells with cathepsin X has been observed, whereas after a longer period of 4 weeks, cathepsin X was present in glial cells, predominantly in activated microglia concentrated in SNc.

Recent studies have demonstrated that lysosomal cysteine cathepsin X might play an important role in neurodegenerative processes, through carboxypeptidase activity within the cells or by release from cells as a proenzyme acting extracellularly, independent of its enzymatic activity (Pišlar et al., 2017; Wendt et al., 2007; Wendt et al., 2009; Hafner et al., 2013; Pislar et al., 2014). In mouse brain, cathepsin X is expressed in almost all cells, with a preference for glial cells, and high levels of cathepsin X have been observed in degenerating brain regions of amyotrophic lateral sclerosis and in AD transgenic mouse models (Wendt et al., 2007; Hafner et al., 2013). Cathepsin X has also been associated with neuroinflammation, as the immunoreactivity for cathepsin X has been shown in some dendritic cells in aged mice brain (Stichel and Luebbert, 2007). Furthermore, a comprehensive comparative gene expression analysis of mouse models of multiple sclerosis, AD, and stroke, found that cathepsin X is one of the 18 genes whose expression is increased in all three models of neuroinflammation (Tseveleki et al., 2010). Additionally, Allan et al. (2017) showed that mice deficient in cathepsin X have reduced neuroinflammation and dramatically lowered circulating levels of interleukin 1β during experimental autoimmune encephalomyelitis, supporting *in vitro* studies of novel role of cathepsin X in neuroinflammation (Pišlar et al., 2017). Nevertheless, other experimental models for neurodegenerative diseases, such as for PD, have described the upregulation of the other lysosomal peptidases [reviewed in Pislar and Kos (2014)]. As such, increased expression of cathepsin L in DA neurons in ipsilateral SNc of a rat PD model, as well as in PD patients, has been reported (Li et al., 2011). Furthermore, it has been shown that cathepsin L mediates 6-OHDA-induced apoptotic events leading to neurodegeneration (Xiang et al., 2011). 6-OHDA, a common neurotoxin used in animal models of PD, also increased the expression of cathepsin B, although inhibition of this enzyme failed to protect neuronal cells (Lee et al., 2006). Nevertheless, *in vitro* study revealed that cathepsin X promotes 6-OHDA-induced apoptosis and the consequent neuronal toxicity, and cathepsin X inhibition exerts neuroprotection of dopaminergic-like neuronal cells (Pislar et al., 2014). Cathepsin X could therefore be involved in neurodegenerative disorders resulting from progressive loss of dopaminergic neurons.

Injection of 6-OHDA into the MFB results in rapid uptake of the toxin by DA neurons, and a near complete loss of the DA neurons in the SNc. After time, there is extensive DA depletion of SNc target areas on the site of injection, including the striatum (Torres and Dunnett, 2011). In our study, 6-OHDA unilaterally induced dopaminergic neuronal degeneration in a time-dependent manner, as indicated by the reduction of TH mRNA hybridization signal in the ipsilateral SNc and VTA. We also observed a reduction of TH-immunoreactivity in the ipsilateral SNc at all times after injection, demonstrating an obvious dopaminergic neuronal degeneration, which is necessary for a successful PD animal model. The time-course of loss of the TH immunosignal in SNc cell bodies is consistent with the study of Jeon et al. (1995), where neuronal death was observed in SNc as early as 12 h after nigral 6-OHDA injection, and prior to striatal terminal degeneration. By *in situ* hybridization, we obtained pronounced upregulation of cathepsin X mRNA levels in the ipsilateral SNc and VTA region, 12 and 24 h after 6-OHDA-induced lesion, whereas, after 48 h, a decreasing trend in cathepsin X mRNA level in ipsilateral SNc was observed. Using immunohistochemistry, we were able to confirm cathepsin X upregulation in the ipsilateral SNc compared to regions in the contralateral sides, as the protein expression pattern was similar to the mRNA pattern at all three time points used for lesion. Additionally, cathepsin X protein level and activity in contralateral and ipsilateral SNc were determined and the values obtained for protein expression correlated well with microscope observation. Interestingly, the cathepsin X activity was significantly altered, but only slightly increased in ipsilateral SNc at 12 and 24 h after injection into MFB.

There are no reports in the literature regarding the expression of cathepsins in the striatum after 6-OHDA injection into MFB. In the hemiparkinsonian rat model used in our study, the destruction of nigrostriatal neurons has been observed (Perese et al., 1989; Deumens et al., 2002). We found no difference in intensity of TH-immunoreactive staining in the ipsilateral striatum, even after 48 h of 6-OHDA-induced lesion, which is in line with other reports of a delay in the degeneration of the striatal DA terminals (Barneoud et al., 1995; Jeon et al., 1995; Ben et al., 1999). Therefore, the results obtained by immunohistochemical analysis, providing similar intensity of cathepsin X-immunoreactivity signals in contralateral and ipsilateral striatum at 12, 24, and 48 h after 6-OHDA, were expected.

Mislocalization and altered secretion of the lysosomal cathepsins have been extensively reported for several pathological conditions [reviewed in Pislar and Kos (2014)]. In AD, some lysosomal proteases are secreted from cells and accumulate extracellularly in the plaque (Cataldo and Nixon, 1990; Cataldo et al., 1991). Similarly, secreted cathepsins have been shown to be a causative factor of inflammation-induced neurodegeneration (Nakanishi, 2003; Wendt et al., 2009; Czapski et al., 2010; Fan et al., 2012). In this work, we have provided evidence that increased cathepsin X in ipsilateral SNc is located predominantly in vesicular structures within the cells, in lysosomes, suggesting that it functions in neurodegenerative processes as a proteolytic enzyme. The upregulation of cathepsin X in ipsilateral SNc is apparently a result of an increased number of cathepsin X-immunopositive cells, as well as its increased cell levels. Within cathepsin X positive cells in ipsilateral SNc, we noted a large proportion of neuronal cells. Specifically, the majority of these cells were dopaminergic neuronal cells and TH-positive cells with increased cathepsin X were indeed strongly increased in the ipsilateral SNc in comparison to the contralateral side.

Although we were able to show that at early time points the upregulated cathepsin X was seen predominantly in the dopaminergic neurons in the ipsilateral SNc, a few microglial cells also showed a positive signal for cathepsin X expression, whereas astrocytes were devoid of cathepsin X. This profile was observed 12 h after injection of 6-OHDA into the MFB; however, cathepsin X-positive microglial cells increased with the prolonged time-period of 6-OHDA-induced lesion. Following CNS injury, activated microglia appear in the SNc as early as day’s 1–3 post-6-OHDA lesioning, and the rapid microglia response is followed by an astrocytic reaction (Marinova-Mutafchieva et al., 2009; Haas et al., 2016). Thereafter, activated microglia release the pro-inflammatory factors that aggravate the loss of dopaminergic neurons (Chen et al., 2005, 2008). The microglial response to neuronal damage is believed to be long-lived and self-propelling (Huh et al., 2003; McGeer et al., 2003). Although the activation of microglia precedes the substantial loss of TH-positive neurons in the midbrain, the death of neuronal loss also contributes to the long lasting microglial activation (Marinova-Mutafchieva et al., 2009; Yokoyama et al., 2011). On the other hand, it has been reported that dopaminergic neurons precede the cell degenerative processes in the SNc and that the microglial activation was strictly concomitant with the neuronal degeneration, which appeared to be a secondary, rather than a primary, phenomenon associated with the loss of dopaminergic neurons (Henry et al., 2009).

Nevertheless, after longer period of time from 6-OHDA injection into the MFB, no neuronal cells were observed at the ipsilateral SNc after 4 weeks of injection, presumably due to complete loss of dopaminergic neurons. On the contrary, microglial reactivity increased and was followed by an astrocyte reaction. Therefore, no neuronal cells positive for upregulated cathepsin X were present in the area of damaged SNc, but dense accumulation of cathepsin X-immunoreactive signals were localized in microglial cells as well in some astrocytes concentrated in the ipsilateral SNc, which correlated well with strongly increased glial reactivity. These results suggest the important role of cathepsin X in processes at an early stage of neurodegeneration, as well in processes in progressive inflammation, which both lead to chronic neurodegenerative disorder resulting from progressive loss of dopaminergic neurons in the SNc. These results are also in line with those of our recent *in vitro* study, where increased cathepsin X expression and activity in microglial cells resulted in microglia activation-mediated neurodegeneration (Pišlar et al., 2017).

## Conclusion

We present, for the first time, the distribution and cellular localization of cathepsin X in the striatum and SNc of 6-OHDA-lesioned rats. 6-OHDA injection into the MFB increased cathepsin X expression and its activity in the SNc at the ipsilateral side, with the simultaneous degeneration of dopaminergic nigrostriatal neurons. The prominent cathepsin X upregulation was restricted to dopaminergic neuronal cells at an early time points after the injection, whereas a late time point of 6-OHDA-induced lesion caused the upregulation of cathepsin X restricted to the glial cells concentrated in the ipsilateral SNc. Based on our previous *in vitro* studies and on the current results, we can propose that cathepsin X is an important causative factor in neurodegenerative processes, resulting in progressive loss of dopaminergic neurons, and is therefore designated as a potential target for therapeutic interventions in PD.

## Author Contributions

AP and MŽ designed the study. AP prepared coronal sections of early time point of injection, performed immunohistochemistry, ELISA assay, cathepsin X activity assay and double immunofluorescence labeling, generated the data for Figures, and prepared the draft manuscript. LT prepared coronal sections of late time point of injection, performed *in situ* hybridization histochemistry, participated in carrying out the immunohistochemistry and double immunofluorescence analysis, and reviewed the manuscript. GG participated in carrying out the immunohistochemistry and reviewed the manuscript. MŽ performed unilateral 6-OHDA lesions. MŽ and JK coordinated the research and reviewed the manuscript. All authors read and approved the final manuscript.

## Conflict of Interest Statement

The authors declare that the research was conducted in the absence of any commercial or financial relationships that could be construed as a potential conflict of interest.

## Supplementary Material

FIGURE S1 Expression of TH and cathepsin X protein in the striatum after 6-OHDA injection. Representative immunohistochemical images for TH **(A)** and cathepsin X **(B)** in coronal midbrain sections of striatum from the 12, 24, and 48 h time-points after 6-OHDA-induced lesion. Scale bars = 50 μm.

FIGURE S2 Lysosomal localization of cathepsin X in the 6-OHDA-induced nigrostriatal lesion. Representative images of double immunofluorescent staining of lysosomal marker LAMP1 (green fluorescence) and cathepsin X (red fluorescence) in the ipsilateral SNc after 6-OHDA injection. Nuclei were counterstained with DAPI (blue). Cathepsin X was strongly localized in LAMP1-positive vesicles in the ipsilateral SNc after 12 h **(A)**, 24 h **(B)**, and 48 h **(C)** of 6-OHDA injection, as indicated by white arrows. For each condition, a group of 4 animals (*n* = 4) was analyzed and four sections of the SNc region of each animal were analyzed. Scale bars = 50 μm.

FIGURE S3 Immunohistochemical analysis of microglia activation in the SNc after 6-OHDA injection. Representative images of double immunofluorescence staining of the microglial marker OX-6 (green fluorescence) and cathepsin X (red fluorescence) in the ipsilateral SNc at 24 h **(A)** and 48 h **(B)** after 6-OHDA-injection. Nuclei were counterstained with DAPI (blue). Scale bar = 50 μm.

## Abbreviations

6-OHDA: 6-hydroxydopamine
BSA: bovine serum albumin
CNS: central nervous system
DA: dopamine
KPBS: potassium phosphate buffer
MFB: medial forebrain bundle
PBS: phosphate buffer saline
PD: Parkinson’s disease
SNc: substantia nigra pars compacta
TH: tyrosine-hydroxylase
VTA: ventral tegmental area
