# Supplementary figures and images for: Upregulation of Cysteine Protease Cathepsin X in the 6-Hydroxydopamine Model of Parkinson’s Disease
Source: Front Mol Neurosci. 2018 Nov 2;11:412. doi: 10.3389/fnmol.2018.00412 (PMC6225071; doi:10.3389/fnmol.2018.00412)

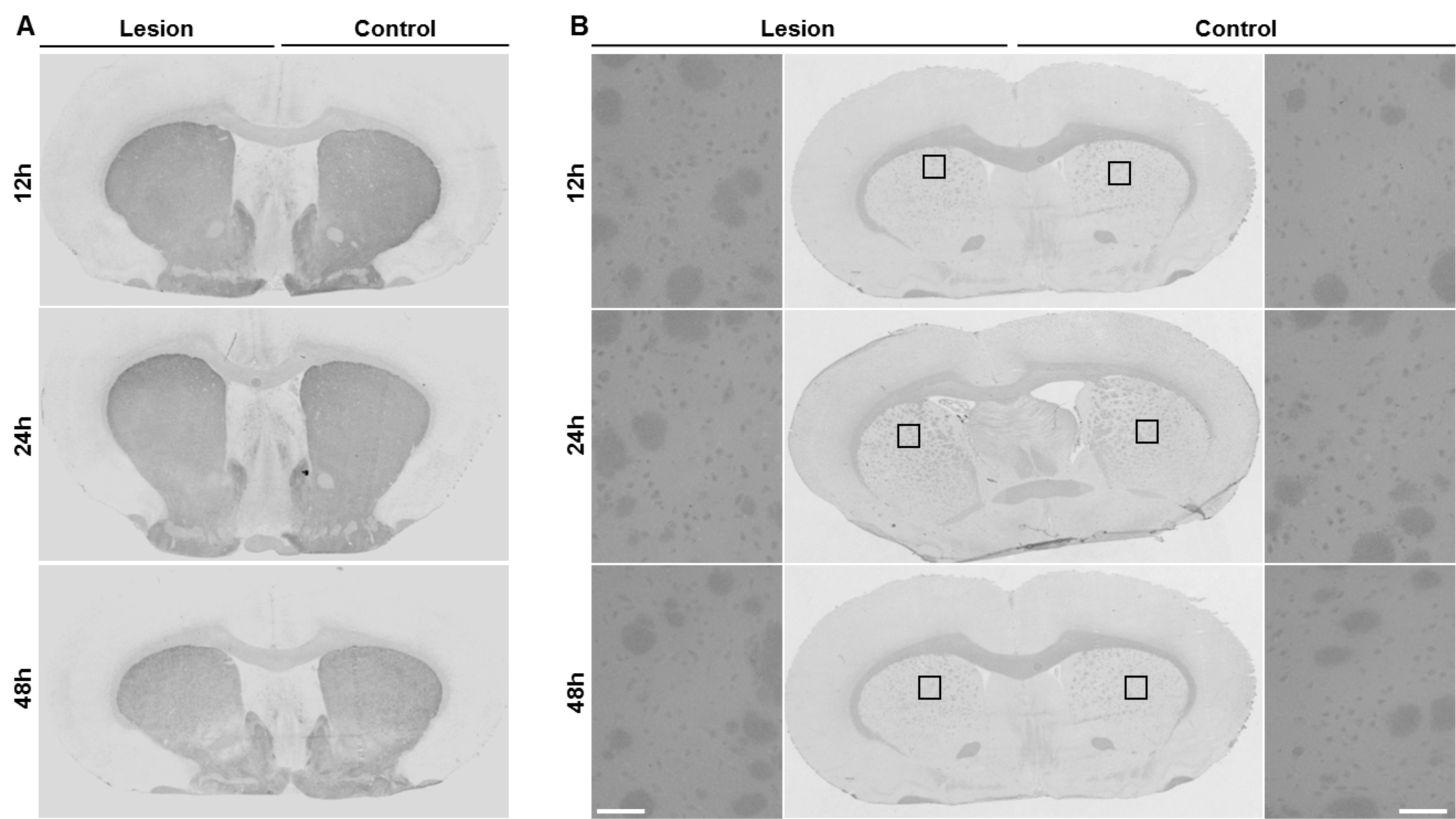

Supplement: FIGURE S1 — Expression of TH and cathepsin X protein in the striatum after 6-OHDA injection. Representative immunohistochemical images for TH (A) and cathepsin X (B) in coronal midbrain sections of striatum from the 12, 24, and 48 h time-points after 6-OHDA-induced lesion. Scale bars = 50 μm. [file Image_1.TIF]

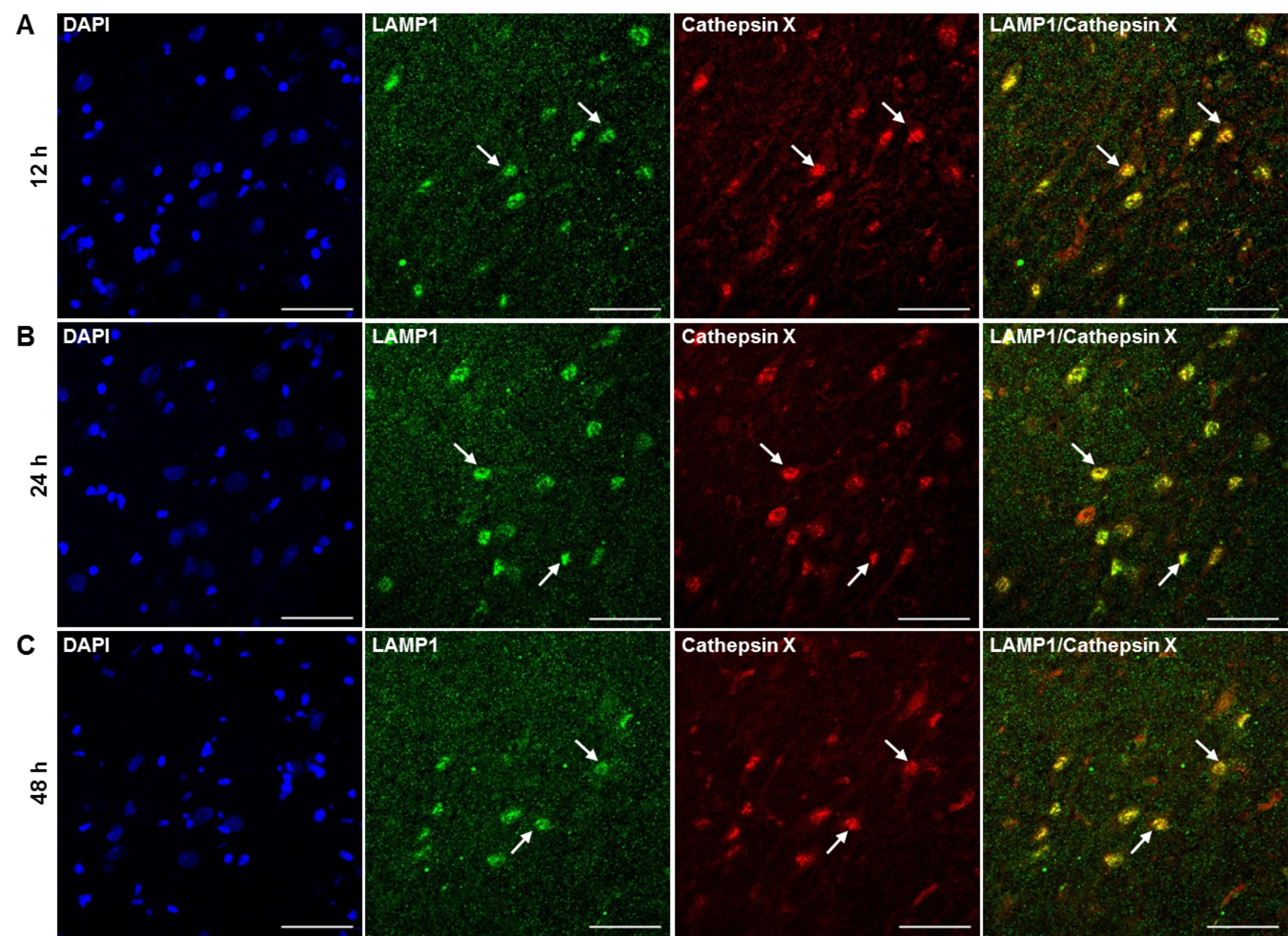

Supplement: FIGURE S2 — Lysosomal localization of cathepsin X in the 6-OHDA-induced nigrostriatal lesion. Representative images of double immunofluorescent staining of lysosomal marker LAMP1 (green fluorescence) and cathepsin X (red fluorescence) in the ipsilateral SNc after 6-OHDA injection. Nuclei were counterstained with DAPI (blue). Cathepsin X was strongly localized in LAMP1-positive vesicles in the ipsilateral SNc after 12 h (A), 24 h (B), and 48 h (C) of 6-OHDA injection, as indicated by white arrows. For each condition, a group of 4 animals (n = 4) was analyzed and four sections of the SNc region of each animal were analyzed. Scale bars = 50 μm. [file Image_2.TIF]

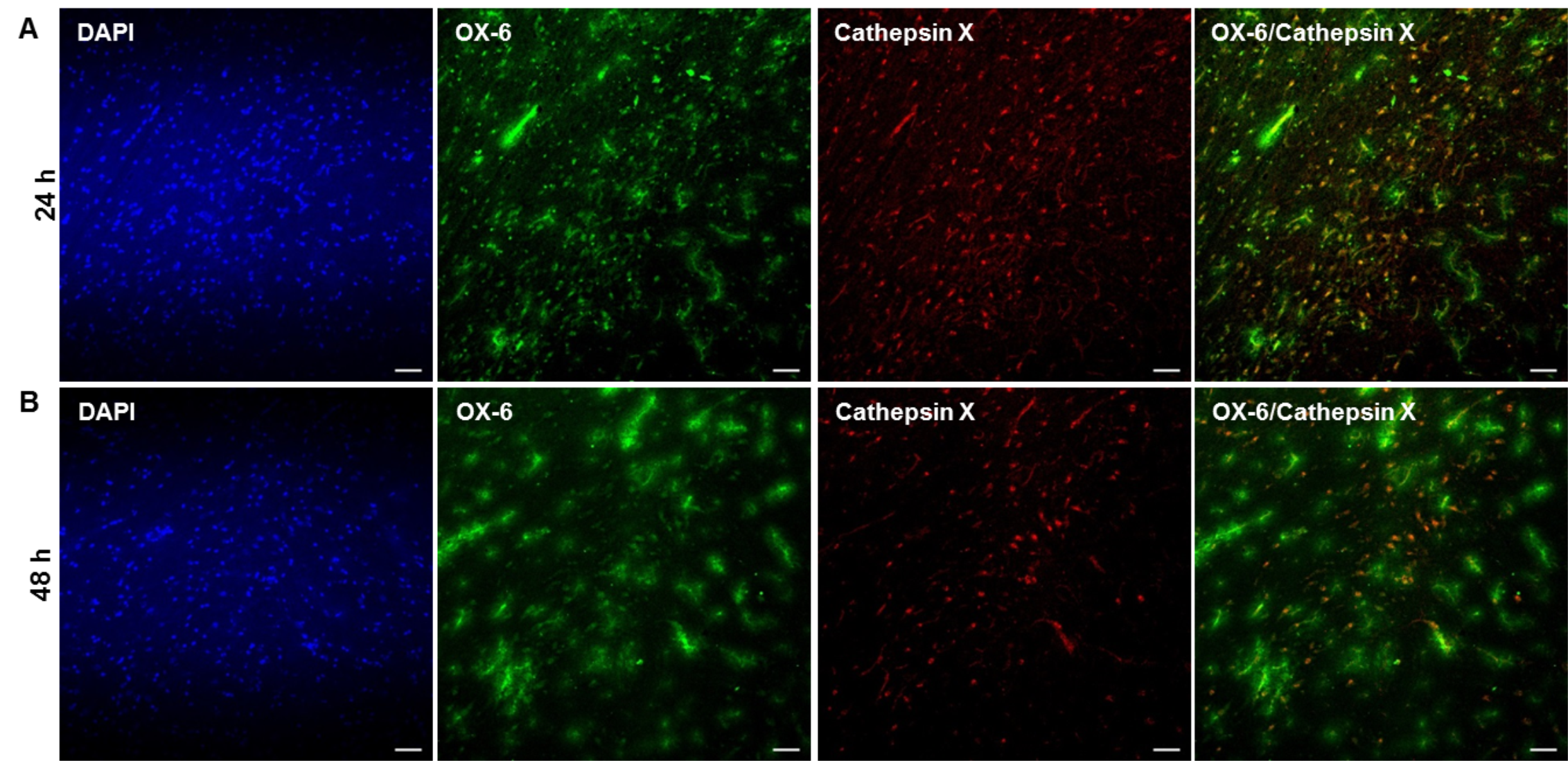

Supplement: FIGURE S3 — Immunohistochemical analysis of microglia activation in the SNc after 6-OHDA injection. Representative images of double immunofluorescence staining of the microglial marker OX-6 (green fluorescence) and cathepsin X (red fluorescence) in the ipsilateral SNc at 24 h (A) and 48 h (B) after 6-OHDA-injection. Nuclei were counterstained with DAPI (blue). Scale bar = 50 μm. [file Image_3.TIF]
